# Development of a Silicone-Based Polymer Matrix as a Suitable Transdermal Therapeutic System for Diallyl Disulfide

**DOI:** 10.3390/ph15101182

**Published:** 2022-09-23

**Authors:** Szabolcs László, Zsófia Hajna, Attila Egyed, Erika Pintér, Ödön Wagner

**Affiliations:** 1 Department of Pharmacology and Pharmacotherapy, Medical School, University of Pécs, Szigeti u. 12., H-7624 Pécs, Hungary; 2 Department of Inorganic and Analytical Chemistry, Faculty of Chemical Technology and Biotechnology, Budapest University of Technology and Economics, Műegyetem rkp. 3., H-1111 Budapest, Hungary; 3 Molecular Pharmacology Research Group, Szentágothai Research Centre, University of Pécs, Ifjúság ú. 20., H-7624 Pécs, Hungary; 4 Medicinal Chemistry Research Group, Research Centre for Natural Sciences, Magyar Tudósok Körútja 2., H-1117 Budapest, Hungary

**Keywords:** diallyl disulfide, hydrogen sulfide, TRPA1 receptor, transdermal therapeutic systems, silicone, membrane diffusion

## Abstract

There is an unmet need for novel therapeutic tools relieving chronic pain. Hydrogen sulfide (H_2_S) is highly involved in pain processes; however, the development of ideal matrices for sulfide donor compounds remains a great pharmaceutical challenge. We aimed to establish a suitable transdermal therapeutic system (TTS) using the H_2_S donor diallyl disulfide (DADS) as a model compound. After the preparation of DADS, its solubility was investigated in different liquid excipients (propylene glycol, polyethylene glycol, silicone oil) and its membrane diffusivity was assessed in silicone matrices of different compositions. Drug-releasing properties of DADS-containing patches with different silicone oil contents were determined with Franz and flow-through cells. We found a correlation between the liquid excipient content of the patch and the diffusion rate of DADS. DADS showed the best solubility in dimethyl silicone oil, and the diffusion constant was proportional to the amount of oil above the 3 m/m% threshold value. The 8-day-old patch showed a significantly lower, but better-regulated, drug release over time than the 4-day-old one. In conclusion, the silicone-based polymer matrix developed in this study is suitable for stable storage and optimal release of DADS, providing a good basis for a TTS applied in chronic pain.

## 1. Introduction

Chronic pain, affecting one fifth of the world’s population [1,2], is often caused by neuropathic states and inflammatory conditions of various etiology [3,4,5].

Hydrogen sulfide (H_2_S) is a ubiquitous gasotransmitter, participating in numerous physiological processes, such as pain modulation and inflammation [6]. In order to achieve a proper antinociceptive effect, H_2_S has to be released slowly from any sulfide donor molecule [7]. Therefore, the potential clinical application of H_2_S and its donor compounds is in the focus of modern medicine [8]. However, the lack of optimal carrier systems being able to store and liberate slow-release sulfide donor molecules remains a challenging pharmaceutical purpose mainly because of short plasma half-life, poor stability, and inconvenient routes of administration [8,9].

Sulfide donor molecules can be divided into naturally occurring organosulfur compounds (OSCs) and synthetic H_2_S donors [8]. Natural OSCs include the pharmacologically active constituents of garlic (*Allium sativum*), such as the highly reactive allicin and its allyl sulfide derivatives. Allyl sulfides are remarkably more stable than allicin and are able to release H_2_S under physiological conditions [10,11]. Moreover, garlic-derived OSCs show structural similarities with allyl isothiocyanate (AITC, found in mustard oil) and they are able to excite an AITC-sensitive subpopulation of peptidergic primary sensory neurons via the activation of the Transient Receptor Potential Ankyrin 1 (TRPA1) receptor [12].

Among the allyl sulfide compounds of garlic, diallyl disulfide (DADS) shows numerous chemical, physiological, and commercial advantages. First of all, DADS is a natural compound that is readily available, easily reproducible, and cheap. Moreover, it is also stable and can be regarded as a slow-release H_2_S donor [13]. Regarding the biological functions, it has been shown to exert a number of beneficial effects, e.g., anti-inflammatory, antioxidant, antinociceptive, and neuroprotective effects, among others [14,15]. In animal experiments, orally or intraperitoneally administered DADS attenuated traumatic neuropathic and chronic inflammatory pain [16,17]. However, in the clinical practice it is highly essential which route of administration provides the most effective and most comfortable treatment for the patient, raising the necessity of potential transdermal application of sulfide-donor molecules. Since H_2_S has to be released slowly from its donor compounds in order to achieve analgesic effect [7], the establishment of an appropriate transdermal therapeutic system (TTS) is needed for carrying and liberating slow-release sulfide donor molecules. Because of its aforementioned advantageous characteristics, DADS was chosen as a model compound for this purpose.

The topical administration of DADS requires the selection of a suitable carrier. The simplest method is the application of crushed garlic to the painful area, but a slightly more advanced method is to apply it in the form of an ointment. However, this has several drawbacks: the ointment stains clothing and has a strong odor (due to the volatility of DADS and related compounds) which can cause discomfort.

Since the amount and rate of delivery of the active product is not well controlled by either method, it is necessary to develop some kind of stable carrier for the active substance. For this purpose, the use of a pharmaceutical patch form is almost self-evident. The most commonly used patch forms are designed to carry drug molecules of some polar character; however, DADS has a highly apolar, hydrophobic character [18]. As a solution, we chose to prepare the carrier system from a poly-dimethyl siloxane polymer matrix, which is rarely used for this purpose [19].

The TTSs provide an excellent mode of accurate, safe, and painless dosing in drug therapy. Dermal absorption systems can be categorized on the basis of their structure or their chemical composition [20]. According to the structure, they can be single- or multilayer adhesive polymer dispersion-based type [21], membrane-controlled type, polymer-matrix-controlled diffusion type, “micro-reservoir” type systems, and a novel technology of microfabricated microneedles [22]. Based on their material there are hydrophilic organic copolymers (e.g. polyols, polyethers, etc.) and silicone-based systems (hydrophobic or modified amphiphilic structure). However, currently established polymers (e.g., polyether, polyurethane) are unsuitable because the essentially reactive sulfides can interact with the matrix structure, degrading the active substance itself as well as the carrier polymer.

From the point of view of drug release, membrane-controlled systems possess the most favorable characteristics. Their only disadvantage is that the drug is in the liquid phase under the control membrane, so the patch cannot be cut and the dose rate cannot be varied. Adhesive polymer dispersion systems are excellent in this respect, but the kinetic of drug release is suboptimal. The two characteristics are well combined in “micro-reservoir” type systems, but these are expensive to produce. This type of TTS is thicker than others and release is controlled by diffusion through the polymer matrix [23]. The construction of the polymer matrix from organic polymers is complicated due to solubility and other chemical properties [24].

**Figure 1:**
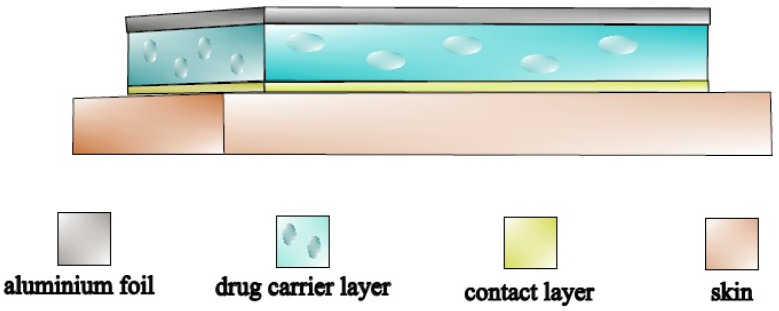
Modified silicone-based polymer matrix-controlled diffusion TTS.

Modified silicone-based polymer systems have been developed by our workgroup [25,26], and provide well-controlled and cost-effective matrix diffusion systems (Figure 1). The basis of silicone polymers is hydroxyl terminated dimethylpolysiloxane, a linear organosilicon polymer which can be cross-linked to form elastic silicone rubber. We used this material as support matrix. In polymers produced by condensation, Si–O–Si bonds are the cross-links. The structure of addition polymers is difficult to modify, whereas the cross-linked structure of condensation polymers is more easily altered. The final matrix structure can be easily modified with different additives to improve the mixing of the active ingredient and the matrix. The condensing silicone polymer can be prepared from two main components: linear hydroxyl terminated dimethylpolysiloxane and tetraethoxy-silane [27].

The optimal composition of the matrix is clearly indicated by the diffusion properties of the drug in the matrix, which can be well described by the diffusion constant. Based on the literature, matrices containing different amounts of apolar excipients (silicone oil) were investigated and possible compositions were selected, on the basis of which the experimental patches were prepared [28,29].

The active ingredient and adjuvants are incorporated into the polymer of the TTS. The viscous mixture cures in about 30–60 min at room temperature, depending on the composition. A carrier layer is required due to poor mechanical properties of the silicone polymer. Usually, a metal or polymer film is utilized. The polymer mixture can be applied to the carrier layer by a single-layer spreading method.

Based on these data, the present study aimed to develop and investigate a silicone-based polymer matrix that is suitable for the application of the slow-release H_2_S-donor DADS in a transdermal therapeutic system. The main objective of our experiments was to find a suitable carrier for the promising sulfide-containing active substances.

## 2. Results

### 2.1. Solubility of DADS

In order to find the most suitable adjuvant, we investigated the solubility of DADS in different materials. For this study we selected PG, PEG 400, and M-350, which were approved for medical application. The best solubility (0.4 g DADS/g silicone oil) was detected in dimethyl silicone oil, lower solubility was measured in PEG400 (0.3 g DADS/g PEG400), and the lowest solubility was observed in PG (0.05 g DADS/g PG). These results clearly indicate the superiority of the silicone oil over the organic materials.

### 2.2. Results of Membrane Diffusion Measurement

**Figure 2:**
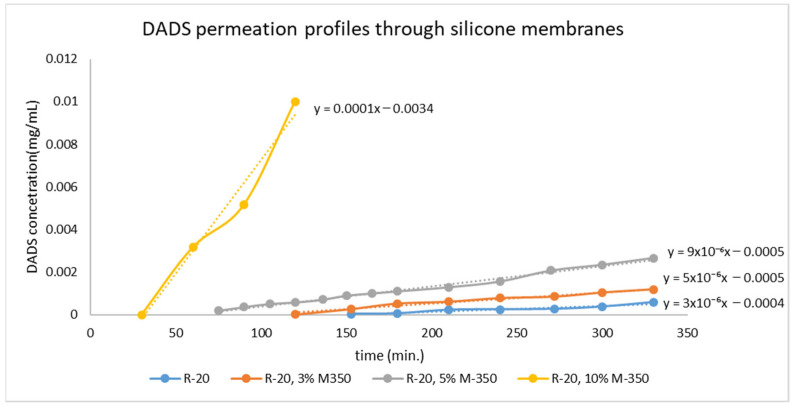
DADS permeation through different silicone membranes.

**Figure 3:**
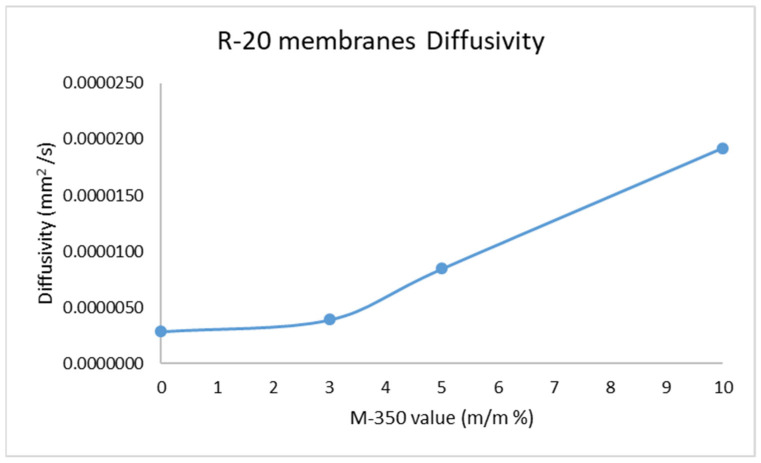
Effect of filler value on the DADS diffusivity through the silicone membranes.

After the selection of the proper adjuvant, the preparation of the membrane was carried out, and the diffusion of DADS was investigated (Figure 2). We found that the diffusion constant of the drug increases with increasing amount of silicone oil (Table 1). Based on the measured data, a silicone oil content of 3% is the threshold value; up to this amount of excipient, there is barely a change in the diffusion properties of the drug in the matrix. However, above this amount, the change in the diffusion constant is proportional to the increase in the amount of oil (Figure 3).

### 2.3. Investigation of Stability of the Drug Release with Franz Diffusion Cell

**Figure 4:**
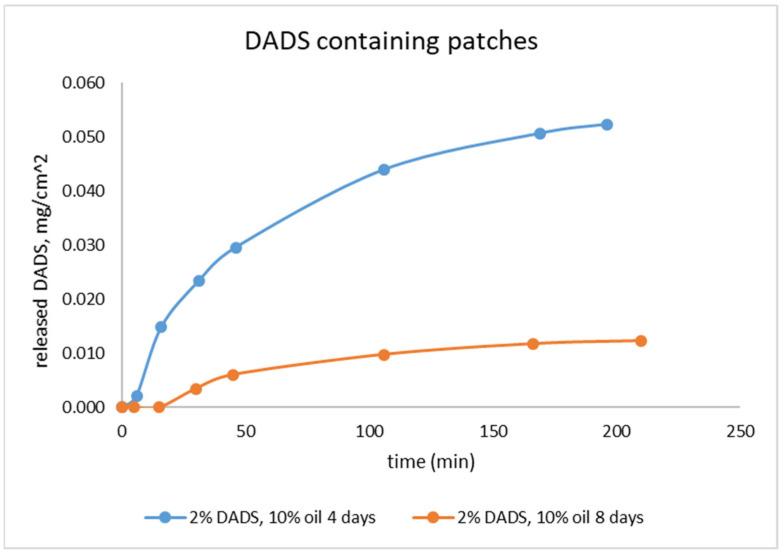
The cumulative release rate of the samples in the Franz cell.

Dermal patches with two different lifetimes (4 and 8 days) were studied with in vitro release test (IVRT) (Figure 4). The cross-linked structure of the sample stabilizes in at least 3 days under preconstruction conditions, based on previous results [25]. Therefore, we investigated the drug delivery properties of the sample on the fourth day. After the same time period, we determined the stability of the delivery, since at this time the change in the drug delivery properties is not related to the change in the matrix structure.

In the IVRT measurement, a substantially larger amount of DADS was released from the 4-day-old patches within 3 h compared to 8-day-old ones. The 8-day-old patches had significantly lower release value. The extent of release (IVRT) itself holds relevant information for comparison based on lifetime [30].

### 2.4. Investigation of Drug Release with Flow-Through Cell

Dermal patches with two different silicone oil content (5 and 10 m/m%) were studied by modified IVRT. In the measurement, substantially larger amount of DADS was released from the 4-day-old patches within 6 h compared to 8-day-old ones.

**Figure 5:**
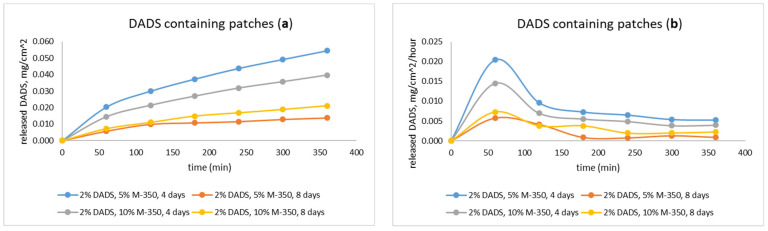
The cumulative (**a**) and continuous (**b**) release rate of the samples in the flow-through cell.

In the flow-through cell, the regulation of drug release of the patches was monitored. The older DADS patch had a better regulated drug release over time, but the released amount was lower (Figure 5).

## 3. Discussion

In this study, we have found a correlation between the diffusion rate of DADS and the liquid excipient content of the patches. The best solubility was achieved with the dimethyl silicone oil, with a 3 m/m% threshold value significantly changing the diffusion properties of the drug in the matrix. In the Franz cell we studied 4- and 8-day-old patches, and the 8-day-old patch had a significantly lower release value. In the flow-through cell, the older patch showed a better regulated drug release over time, but the released amount was smaller.

We have modified the usual recipe of the literature, enabling the production of DADS with better yield and higher purity. The use of a 90% purity material made the interpretation of the leaching assays much easier and clearer [19].

We have also demonstrated that the properties of the compounds in silicones were absolutely necessary to determine whether they were suitable for use in the transdermal formulation. As DADS is an apolar compound, its diffusion in the silicone matrix could be facilitated by the use of an apolar liquid phase compatible with the silicone matrix. The most suitable for this purpose is polydimethylsiloxane oil (M350), in which DADS is very soluble, the saturated solution containing 0.4 g/g DADS.

In membranes containing different concentrations of silicone oil (0%, 3%, 5%, 10%), the diffusion of DADS through the membrane increased with rising amount of silicone oil. The 3% concentration of silicone oil was not enough to allow a continuous liquid phase to form in the matrix, so the diffusion of DADS only slightly increased. In contrast, the 5% concentration of silicone oil led to a threefold increase in the diffusion constant; moreover, the 10% concentration of silicone oil resulted in a tenfold increase.

With these results, we prepared silicone matrices consisting of a single layer of 2% DADS and 5% and 10% M350 silicone oil and investigated its ability to stabilize the volatile DADS content. Our tests were first performed in the Franz cell, as it is widely used for the investigation of transdermal applicable drugs [31,32], with samples containing 10% silicone oil after 4 and 8 days from preparation. IVRT measurements showed that the volatility of DADS was preserved, with samples stored for 8 days having one fifth as much DADS dissolved as samples stored for 4 days. Figure 4 clearly shows that in the case of the 4-day-old sample, DADS was detectable in the leachate after 10 min, whereas in the case of the 8-day-old sample it was only detectable after 30 min. During storage, the volatile active ingredient is continuously removed from the matrix of the formulation, mainly from the layers close to the surface, which becomes increasingly difficult to replace from the deeper layers as time goes on. Our results show that the readily releasable drug content of the patch was reduced by 80% in 4 days, which highlights the necessity of the investigation of further other sulfide donor compounds for this purpose.

We also investigated the release of the active substance using our flow cell model, as it gives a better model for the drug release during patch application. The fluid that passes in front of the sample surface models the blood flow that carries the drug to the tissues of the body. In this case, the drug content of the fluid can be considered to be zero at all times, so the concentration gradient of the drug is not theoretically decreasing, but is considered to be constant.

These results are in accordance with those gained with the Franz cell experiments. The dissolution of the active substance has a similar course for all samples. For the 4-day-old samples, the loss of active ingredient in the 10% silicone oil samples is greater than in the 5% samples, so that more active ingredient is released from the 5% silicone oil samples. The drug loss increases further during storage, so that the amount of DADS remaining in the samples will be almost the same in both samples (5% and 10% silicone oil), so that the dissolution curves almost overlap. However, when looking at the cumulative drug release curves (Figure 5a), it can be seen that the samples stored for 8 days had a significant reduction in drug content due to their volatility, but the higher silicone oil content sample still had more DADS dissolved in the silicone oil, so the total amount of drug released from this sample is more than the sample containing 5% M350.

These results show that silicone oil promotes the diffusion of DADS and this increases proportionally with the amount of silicone oil used. However, the volatility of DADS is high, so that the amount of active ingredient decreases steadily with time, and the effect of the amount of silicone oil on the rate of release of the active ingredient is eliminated over time. Further work should definitely address the issue of reducing the volatility of DADS, as our results suggest that the compound would be suitable for use in a transdermal formulation.

Naturally occurring OSCs are able to exert beneficial effects in inflammatory and pain processes, similarly to the biological actions of H_2_S [10,15,30]. Garlic—representing the most important member of the *Allium* family of plants—is regarded one of the richest vegetable in OSCs [33]. Garlic serves as a popular culinary ingredient all over the world and has been widely used as a herbal medicine for many centuries. OSCs of garlic include allicin (diallyl thiosulfinate) that is unstable in aqueous media. Allicin decomposes into its oil-soluble derivatives: diallyl sulfide (DAS), diallyl disulfide (DADS), and diallyl trisulfide (DATS) [8,33]. Allyl sulfides are responsible for the pungency of garlic, they show greater stability than allicin under normal conditions (room temperature, oxygen, and humidity), and are able to liberate H_2_S under physiological circumstances [10,11].

Garlic-derived OSCs, such as DADS, are structurally similar to the TRPA1 receptor agonist AITC, so it is not surprising that they can activate TRPA1 [12]. TRPA1—being a member of the Transient Receptor Potential (TRP) receptor superfamily—is a nonselective cation channel that is highly involved in the mediation of inflammation and pain sensation [34,35,36]. The activation of the receptor occurs following the conjugation with its cysteine residues and the formation of disulfide bridges, similarly to the effect of H_2_S on TRPA1 [37].

As the activation of peptidergic primary afferents via TRP channels and the release of several mediator molecules from these fibers are highly involved in the pathomechanism of chronic pain states, these components of the neurogenic inflammation are undoubtedly in the focus of chronic pain management [5]. Chronic pain means not only a serious individual burden for the patients but also a major problem for public health and society worldwide. Chronic neuropathic pain can occur upon traumatic nerve lesion, diabetes, neurodegenerative disorders, and viral infections, while chronic inflammatory pain often accompanies inflammatory disorders such as osteoarthritis, etc.) [1,2,3,4,5].

Recently, DADS was reported to relieve traumatic neuropathic pain in a sciatic nerve ligation model when given orally [16], as well as to attenuate Complete Freund’s Adjuvant-induced inflammatory pain when given intraperitoneally [17]. Similarly, the inhalation of H_2_S, as well as the intraperitoneal injection of the alkyl sulfide molecule dimethyl trisulfide (DMTS), have also been shown to diminish diabetic and traumatic neuropathic pain, respectively [38,39]. The involvement of TRPA1 activator sulfide compounds in nociception, as well as the extensive use of capsaicin—the potent agonist of another TRP channel, the Transient Receptor Potential Vanilloid 1 (TRPV1) receptor—in form of transdermal patches for neuropathic pain [40], raised the question of whether TRPA1-stimulating agents, such as H_2_S and its donor compounds, could be applied in the form of a transdermal therapeutic system. Advantages of transdermal administration are the following: (1) it is suitable for chronic application, (2) continuous and stable plasma level can be achieved, (3) first pass metabolism can be readily bypassed, (4) the administration of the drug can be ceased easily with the removal of the patch, and (5) the dosage can be conveniently modified by cutting the patch.

Since the role of H_2_S in pain modulation and inflammation is influenced by the dose and release rate of application (low-dose H_2_S donors, as well as slow H_2_S releasing agents, may attenuate nociceptive and neuropathic pain, while high-dose and/or fast-released H_2_S acts proinflammatory [6,7,9]), it is crucial to establish an appropriate TTS being able to carry and liberate slow-release sulfide donor molecules. In recent years, a number of slow-release sulfide donor compounds have been developed, such as GYY4137, ADT-OH, AP39, AP67, AP123, and FW1256, as well as thioester-based H_2_S donors [41,42,43,44,45,46,47], raising the possible efficacy of these compounds in chronic neuropathic and inflammatory pain syndromes. Although several efforts have been made in order to enable the transdermal delivery of slow-release H_2_S donors [48,49,50], the establishment of an optimal matrix/carrier system is still an unsolved problem.

Since DADS was proven to release H_2_S in a slow and sustained manner [13], its structure has also served as a useful template for designing other slow-release H_2_S donors [47]; beside its other advantages, it was reasonable to perform the experiments of our study with DADS.

In summary, the results of the present work demonstrate that the chemical inertness of the poly-dimethyl siloxane-based matrix makes it suitable for the delivery of sulfide-based active substances. In addition, DADS serves as an adequate model compound for the development of a transdermal therapeutic system for sulfide-containing substances, thus providing an excellent basis for the future development of transdermal patches with other promising slow-release sulfide donor molecules.

## 4. Materials and Methods

### 4.1. Chemicals

Propylene glycol, polyethylene glycol, sulfur, and sodium sulfide were purchased from Reanal, Hungary. Tetrabutyl ammonium iodide and allyl bromide were purchased from Sigma-Aldrich.

R-20™, condensation cross-linkable polydimethylsiloxane-(α, ω) –diol, Oxam™ catalyst, and M350 polydimethylsiloxane oil were from T-Silox Ltd., Hungary.

All reagents and materials were used without purification.

### 4.2. Instrumental

IR spectra were recorded on a Perkin Elmer Spectrum Two FTIR instrument with a UATR head. ^1^H NMR spectra were recorded in CDCl_3_ solution at room temperature, on a Varian Unity Inova 500 spectrometer (500 MHz for 1H NMR spectra), with the deuterium signal of the solvent as the lock and TMS as the internal standard. Chemical shifts (d) and coupling constants (*J*) are given in ppm and Hz, respectively. Mass spectra (MS) were obtained by a GC/MS QP-2010 spectrometer (EI, 70 eV). Analysis of diallyl disulfide was carried out on a GC/MS QP-2010 spectrometer. The capillary column used was ZB5-MSI, 30 m in length and 0.25 mm in diameter. Conditions were as follows: column temperature began at 80 °C, then increased to 250 °C; helium was used as carrier gas at a linear flow of 1 mL/min. HPLC-MS measurements were performed using a Shimadzu LCMS-2020 device equipped with a Reprospher 100 C18 (5 µm; 100 × 3 mm) column and positive-negative double ion source (DUIS±) with a quadrupole MS analyzer in a range of 50–1000 *m/z*. Sample was eluted with gradient elution using eluent A (0.1% formic acid in water: acetonitrile 19:1) and eluent B (0.1% formic acid in water: acetonitrile 1:19). Flow rate was set to 1 mL/min. The initial condition was 0% B eluent, followed by a linear gradient to 100% B eluent by 1 min, from 1 to 3.5 min 100% B eluent was retained; and from 3.5 to 4.5 min, back to initial condition with 5% B eluent and retained to 5 min. The column temperature was kept at room temperature and the injection volume was 1 µL.

### 4.3. Production of Diallyl Disulfide

First, sodium disulfide solution was prepared by sulfur and sodium disulfide as follows: 6.4 g (0.2 mol) sulfur and 48 g (0.2 mol) sodium sulfide were dissolved in 150 mL distilled water in a round bottom flask. The solution was stirred and heated at 60 °C in one hour, and after that it was cooled to room temperature and filtrated in paper filter. To the brownish-red solution, 0.5 g tetrabutyl-ammonium iodide was added as phase transfer catalyst. A total of 36.3 g (0.3 mol) allyl bromide was added dropwise in 20 min and the temperature of the reaction mixture increased. After addition, the reaction mixture was cooled with ice. The resulting mixture was extracted with 300 mL ether. The organic phase was dried over anhydrous MgSO_4_ overnight. The organic phase was performed on rotary evaporator under aspirator pressure to eliminate the solvent. Then, the residual phase was distilled in low vacuum. The fraction at 45–60 °C and 0.8 torr. was gathered. A total of 25.8 g oil with light yellow color and intensive garlic smell was obtained as the diallyl disulfide with the yield of 88.3%.

The function group was identified by FTIR (UATR, cm^−1^): 3081 (=C–H); 3009 (-CH_2_); 2979 (-CH_2_); 1634 (C=C); 1422 (C–S);

^1^H-NMR (CDCl_3_, 500 MHz): 3.33 (d, 4H), 5.16 (m, 4H), 5.84 (m, 2H).

### 4.4. Production of Silicone Membranes

Membrane samples used in experiments were prepared on a Teflon desk. Silicone oil M350 was dissolved in raw material poly-dimethyl siloxane-(α, ω)-diol R-20. After that, Oxam cross-linker was added to the mixture under stirring. Mixtures were homogenized and spread on the Teflon desk at a thickness of 0.4 mm. The film was cross-linked at room temperature. Samples were rested for 72 h and examined afterward.

The composition of samples was the following:

R20™: 80–90 m/m%.

Oxam™: 10 m/m%.

Silicone oil M-350: 0–10 m/m%.

### 4.5. Production of Diallyl Disulfide Containing Transdermal Patches

TTS samples used in experiments were prepared on a paper substrate laminated on aluminum foil of 0.4 mm thickness. DADS was mixed into silicone matrix carriers. Our raw material was polydimethylsiloxane-(α, ω)-diol R-20™. DADS was dissolved in M350 and was added to the silicone stock. If needed, fumed silica was added, too. After the components were weighed, Oxam™ cross-linker was added to the mixture under stirring. Mixtures were homogenized and spread on the supporting film at a thickness of 0.4 mm. The layer was cross-linked at room temperature. The procedure was finished in 30 min. Samples were rested for 48 h and examined afterwards.

The composition of samples was the following:

R20™: 83%, 78%.

Oxam™: 10%.

Silicone oil M350: 5%, 10%.

DADS: 2%.

### 4.6. Measurement of Membrane Diffusion

The permeation of DADS through the silicone (PDMS) membrane with oil was investigated by using an in vitro membrane permeation system. The thickness and the oil content in the membrane were varied and both the time lag and the steady-state rate of permeation were measured. The membrane was mounted between the two half-cells of the membrane permeation system. A total of 25 mL of phosphate-buffered saline with 25 m/m% ethanol (PBS-E25) without drug was placed into the receptor compartment. A total of 10^−3^ M DADS containing PBS-E25 was added into the donor compartment. At each of the predetermined time intervals, a sample was withdrawn and analyzed by a UV/VIS spectrophotometer (Perkin-Elmer Lambda 25) at 207 nm.

(1) $$D=\frac{l^{2}}{6t_{0}}$$

Equation (1). Diffusivity by the time lag method of Daynes.

The time lag and the steady-state rate of permeation were then determined from the concentration vs. t profiles. The diffusion constants were determined from the time lags using Equation (1) [28].

### 4.7. Measurement of the In Vitro Release of DADS-Containing Transdermal Patches

In vitro testing was performed in two ways. First, we performed an indicative measurement in a Franz cell, which models the static and vertical subcutaneous drug dissolution. In the second method, the patches were examined in a flow-through cell device that mimics the dissolute drug concentration in the blood.

#### 4.7.1. Measurement in the Franz Cell

In vitro release tests (IVRTs) were performed. A modified local fabricated Franz type diffusion cell was used to model the DADS release from the patches in case of IVRT.

The receptor phase was phosphate buffer (PBS pH 7.4 ± 0.15) and 25% *w/w* ethanol 96% at RT. The investigation lasted for 3 h. The stirring speed was 450 rpm. The concentration of the drug was examined by a spectrophotometer (Perkin-Elmer Lambda 25) at 207 nm.

#### 4.7.2. Measurement in the Flow-Through Cell

**Figure 6:**
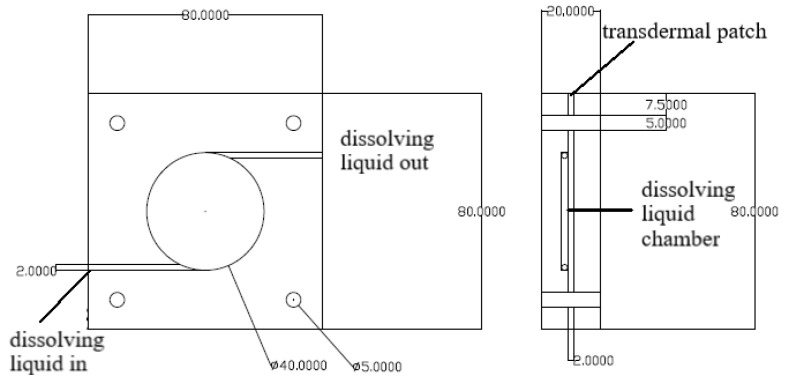
The flow-through cell.

**Figure 7:**
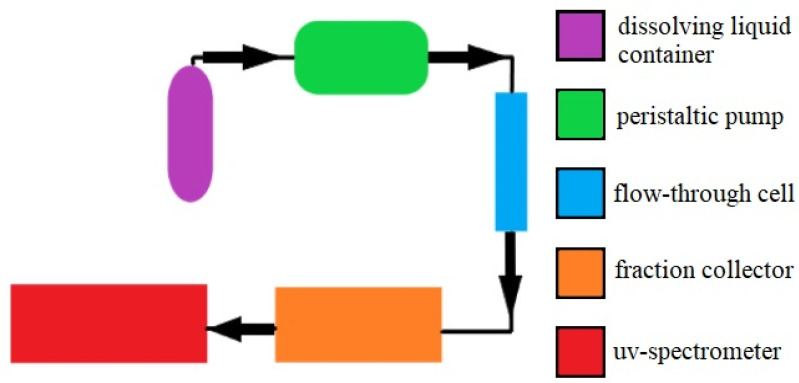
Measurement with flow-through cell.

Samples (12.56 cm^2^ each) of patches were tested for drug release in a flow-through cell at 37 °C ± 0.5 °C (Figure 6). The flow rate (PBS, 25% ethanol) was 25 mL/h, and the DADS content was determined hourly with a spectrophotometer (Perkin-Elmer Lambda 25); the investigation lasted for 6 h (Figure 7).

## Figures and Tables

**Table 1:** Diffusivities and time lags of different silicone membranes.

Sample	l (mm)	t_0_ (s)	D (mm^2^/s)
R-20	0.37	8000	2.85 × 10^−6^
R-20, 3% M-350	0.375	6000	3.91 × 10^−6^
R-20, 5% M-350	0.39	3000	8.45 × 10^−6^
R-20, 10% M-350	0.485	2040	1.92 × 10^−5^

## Data Availability

Data is contained within the article.
